# Advancing fracture management: the role of minimally invasive osteosynthesis in orthopedic trauma care

**DOI:** 10.1007/s00068-024-02634-4

**Published:** 2024-08-21

**Authors:** Florian Wichlas, Gerwin Haybäck, Valeska Hofmann, Amelie Deluca, Andreas Traweger, Christian Deininger

**Affiliations:** 1 Department of Orthopedics and Traumatology, Salzburg University Hospital, Paracelsus Medical University, Salzburg, Austria; 2 No Limit Surgery, Ernest-Thun-Strasse 6, Salzburg, 5020 Austria; 3 Department of Orthopaedic and Trauma Surgery, Karl-Olga-Krankenhaus, Hackstrasse 61, Stuttgart, 70190 Germany; 4 Institute of Tendon and Bone Regeneration, Paracelsus Medical University, Salzburg, Austria

**Keywords:** Minimally invasive, Osteosynthesis, Fracture, Surgical guide

## Abstract

**Purpose:**

Minimally Invasive Osteosynthesis (MIO) developed to be a key technique in orthopedic trauma surgery, offering a less invasive alternative for managing fractures in various anatomical regions. However, standardized guidelines for its application are lacking. This study aims to establish comprehensive principles for MIO to guide surgeons in treating different types of fractures and its locations.

**Methods:**

A retrospective analysis including 57 fractures in 53 patients was conducted. All patients were treated with MIO. Study range - March 2017 to January 2022 at a Level-I trauma university hospital. The analysis covered various fracture types, focusing on surgical approaches, reduction techniques, plate insertion methods, and its outcomes. The efficacy and safety of MIO was evaluated by analyzing complications, fracture healing time, and necessary revision surgeries.

**Results:**

MIO is a versatile and effective fracture treatment that minimized soft tissue damage and ensured stable osteosynthetic results. Articular fractures typically used a “one way up” plate insertion technique, while non-articular fractures applied an “inside-up-and-down” approach. Low complication rates confirmed the safety and efficiency of MIO.

**Conclusion:**

This research established generalized principles for MIO, offering a systematic approach that can be applied for various fracture types and its locations, by overall enhancing the surgical efficiency as well as patient recovery, without compromising outcomes.

**Level of evidence:**

This study is classified as Level III evidence according to “The Oxford 2011 Levels of Evidence”.

## Introduction

### MIO

The application of minimal invasive surgery has steadily increased over the past years [1–3]. In orthopedic trauma surgery, minimal invasive osteosynthesis (MIO) was described for most anatomic regions and its indication is constantly growing ( [4–12]). A MIO approach in fracture care is applicable in every region of the human body. Although MIO was only used sporadically before, the development of locking compression plates (LCP) has enlarged its spectrum of application [13–15]. While the insertion of drill sleeves added another operational step in plate fixation to provide mono axial locking, the sleeves perfectly permit percutaneous locking through stab incisions. Standard approaches usually depend on plate length, but the use of drill sleeves, especially in the distal femur, made this obsolete [16, 17].

In common standard methodologies, the length of the surgical incision permitted all necessary steps for osteosynthesis, ranging from fracture control and reduction, to temporary fixation and plate insertion. In MIO, these steps should be planned independently from each other. Fracture control (visual or digital), reduction using instruments, and plate insertion may all need an individual operative step. In certain scenarios, all steps can be combined in one minimal invasive approach. In the worst case, each step needs an individual single minimal invasive approach.

The advantages and limitations of MIO were discussed extensively in the literature and it can be summarized that it is an effective tool in fracture care [18, 19]. If fractures with severe soft tissue damage, like complex- or open fractures, would undergo a standard operative technique, the risk of soft tissue loss and/or infection would further increase. Therefore, MIO can help limit the damage by placing small incisions into safe zones and by protecting regions that were initially sutured up after an open fracture occurred [20]. The same concept applies for osteoporotic bone fractures [9, 21].

General guidelines for the application of MIO remain scarce in the existing literature. In order to establish these guidelines, we conducted an analysis of MIO-treated fractures across various anatomical regions. The objective of this study was to delineate universally applicable principles for MIO according to diverse fracture types across different anatomical regions. The aim of this research is to provide a comprehensive framework for MIO surgical procedures by having a minimal incision size for a standard, stable osteosynthesis.

## Methods

Between 03/2017 and 01/2022, 57 fractures of 53 patients (mean age 59.94 years) treated with MIO were identified at a Level-I-trauma university hospital in Salzburg, Austria. A positive ethical approval was granted by the Ethics Committee of the State of Salzburg (EK No: 1138/2023). Informed consent has been obtained.

Fractures of different anatomic regions were analyzed to determine general rules and principles of MIO (Table 1).

Inclusion criteria:

- Fractures treated with MIO.
- Articular and non-articular fractures.
- Fractures treated with LCP.
- Closed and open fractures.
- Comminuted and simple fractures.
- Periprosthetic fractures.
- Geriatric fractures > 65 years.

Exclusion criteria:

- Fractures treated with intramedullary nails or K-wires.
- Fractures treated with open reduction and internal fixation (ORIF).
- Spinal and pelvic fractures.
- Patients younger than 18 years of age.
- Pregnancy.

**Table 1:** Patients demographics

	*N*
**Patients**	**53**
Encountered overall fractures	57
-Articular fractures	33
-Non-articular fractures	24
-Patients with 2 different fractures	2
-Patients with lower extremity fractures in the tibia and fibula*	2
-Fractures in geriatric patients (> 65 years old)	22
-Open fractures	5
-External fixation prior to surgery	11
Left Side	18
Right Side	39
Male	24
Female	28

*Patients with fractures in the lower extremity were mentioned separately, as the fractured bones were evaluated separately

The main parameters for MIO evaluated were the surgical approach, the reduction used, and the insertion of the plates.

Secondary parameters included patient´s positioning, feasibility, bony healing, and post-operative complications.

### Surgical techniques applied

Various surgical techniques used for articular and non-articular fractures were analyzed.

The surgical approach itself was defined as an incision, other than a stab incision, to lock plates or for the percutaneous application of instruments (reduction clamps, K-wires or percutaneous lag screws). Stab incisions for instruments were recorded separately.

Surgical approaches used included fracture control, control of the articular surface, plate insertion, or an additional incision for reduction. Therefore, these approaches were evaluated according their location and further divided into four categories:

Surgical approaches:

- Above the articular surface and fracture.
- Above the fracture.
- For plate insertion.
- Additional approaches (for reduction).

### Reduction and temporary fixation

Closed reduction techniques were applied in all fractures. The reduction technique itself was a reduction using the plate as a template by temporary fixation with K-wires through drill sleeves.

Instruments used for reduction and temporary fixation included: Reduction clamps used percutaneously or through the surgical approach, K-wires used percutaneously or through the approach, mini-plates, and cerclage wires.

The reduction techniques used differ depending on the anatomical region and are described in detail below.

MIO reduction technique for articular fractures: The aim is the anatomical reduction of the articular surface, which is why an incision above the joint is necessary in order to be able to inspect and palpate it. After the incision, reduction is performed using reduction forceps and K-wires. Care must be taken to ensure that these do not interfere with the planned plate osteosynthesis. Once the articular surface has been reduced, the previously selected plate osteosynthesis is inserted via the MIO incision remote from the joint and the plate is aligned with the aid of drill sleeves fixed in the fixed-angle holes. The screw holes are then drilled as standard using the familiar ORIF principles but via stab incisions, only. After fixation of the plate osteosynthesis, the reduction forceps and the K-wires are removed.

MIO reduction technique for non-articular fractures: The aim is to restore the correct length, rotation and axes. An incision over the fracture is not compulsory, but can be helpful for reduction. Primarily, the plate bed is created with a raspatory and the selected plate osteosynthesis is inserted via the MIO incision. The plate is aligned with the aid of drill sleeves fixed in the fixed-angle holes. The screw holes are then drilled as standard using the familiar ORIF principles bute via stab incisions, only.

### Plate insertion

Two methods for plate insertion were identified and hence evaluated: Either the plate was inserted “one way up” (or down), or “inside up and down”.

In the “one way up” technique, the plate was inserted through the approach and slid up (or down) along the bony shaft over the fracture.

In the “inside up and down” technique, the plate was inserted through the approach and slid one way up and then the other way down.

Articular fractures occurred mainly in the distal radius, proximal and distal humerus, olecranon, distal femur, and proximal and distal tibia. Non-articular fractures presented in the forearm, clavicle, femur, tibia, distal metaphyseal tibia, and malleolar fibula. The metaphyseal tibia and the malleolar fibula (AO classification code 43 and 44, respectively) were considered as non-articular, due to their intact articular surface. The operated anatomic regions are summarized in Table 2.

**Table 2:** Representation of the operated anatomic regions

		*N*
Articular Fractures	Proximal Humerus	8
	Distal Humerus	6
	Olecranon	1
	Distal Radius	5
	Distal Femur	4
	Proximal Tibia	8
	Calcaneus	1
Non-articular Fractures	Clavicle	7
	Forearm	7
	Femoral Shaft	3
	Tibial Shaft	2
	Distal Tibia	3
	Malleolar (Fibula)	2
Total		57

Eleven fractures were stabilized with an external fixation before MIO and five fractures were classified as open fractures.

Two fractures in geriatric patients (*n* = 22) were initially stabilized using external fixation and included a combined tibial head/shaft fracture and a femoral shaft fracture.

### Surgical technique

Before the first incision, anatomic landmarks were marked on the skin using a sterile pen. If the landmarks could not be palpated safely, the radiological c-arm was used for planning. The goal was to perform a perfect fracture reduction to keep the incisions as small as possible. The orientation of the approaches was chosen according to standard approaches.

The plate bed was made using a raspatory tool before reduction. Plates were inserted and fixed using drill sleeves. The eccentrically inserted drill sleeves were used as a handle to slide the plate under the soft tissues. Two sleeves permitted the manipulation in all directions. The contact of the tip of the plate to the bone could be felt and the plate positioned accordingly. A drill sleeve was inserted in the last plate hole percutaneously and the plate fixed temporarily with K-wires. The position of the plate was confirmed fluoroscopically and adjustments were made if necessary.

A guiding arm for plate insertion was used for the femoral LCP and the calcaneus plate only.

After insertion and temporary fixation of the plate with K-wires through the drill sleeves, a plate dependent lag screw was used to pull the plate to the bone shaft as necessary. The final locking of the plates was made through the existing approach and percutaneously.

### Postoperative protocol

All fractures were treated postoperatively according to AO guidelines. After the soft tissues recovered, early mobilization was encouraged. All patients with articular fractures received pre- and postoperatively CT scans in addition to prior x-ray diagnostics.

## Results

The location of the applied surgical approaches according to the encountered fractures was summarized in Table 3. Some surgical approaches were combined.

**Table 3:** Placement of surgical approaches according to fracture type

	Total	FAS*	Fracture	Plate	Additional
Articular Fractures	33	33	6	1	5
Non-Articular Fractures	24	0	21	7	2
Total	57	33	27	8	7

*FAS: Approach above the Fracture and the Articular Surface

Within the encountered articular fractures, all surgical approaches were above the articular surface and fracture itself. In non-articular fractures, most approaches were above the fracture. Out of 27 approaches above the fracture, 21 (77.8%) occurred in non-articular fractures.

Analyzing all fractures, including articular and shaft fractures, the surgical approach was placed above the fracture (33 above the articular surface and fracture + 21 above the fracture), except in three shaft fractures (closed reduction). This included 2 extraarticular tibial fractures (1 shaft, 1 metaphysis) and 1 femoral shaft fracture (periprosthetic).

A single surgical approach was used in 38 fractures (66.7%), two surgical approaches in 16 (28.1%) patients, and three approaches in 2 (3.5%) patients.

Plate locking was carried out percutaneously through stab incisions.

All approaches except 2 (3.5%) were incised according to previously established standardized surgical approaches, but were limited in their extend. This was best described for the standard palmar approach of the distal radius, which applies to the same location as the palmar Henry approach [14, 22]. The goal of this minimal invasive approach was to control the fractured palmar cortex, the key landmark for reduction. The 2 exceptions for using limited standard approaches included the distal humerus (*n* = 6; described by Hofmann et al.), and the calcaneus, where an Ollier approach was used (*n* = 1) [23].

Various techniques and instruments used for fracture reduction and temporary fixation was summarized in Table 4. Some of these techniques were combined.

Reduction could be achieved in all fractures except for one, as confirmed by intra- and post-operative x-ray imaging, confirmed by CT scans for articular fractures.

**Table 4:** Summary of fracture reduction techniques

	*N*
Closed Reduction	57
Reduction Clamp	33
K-wire	30
Plate Reduction	29
PC* K-wire	15
PC Reduction Clamp	9
Mini Plates	2
Cerclage	1

*PC = percutaneous

**Fig. 1:**
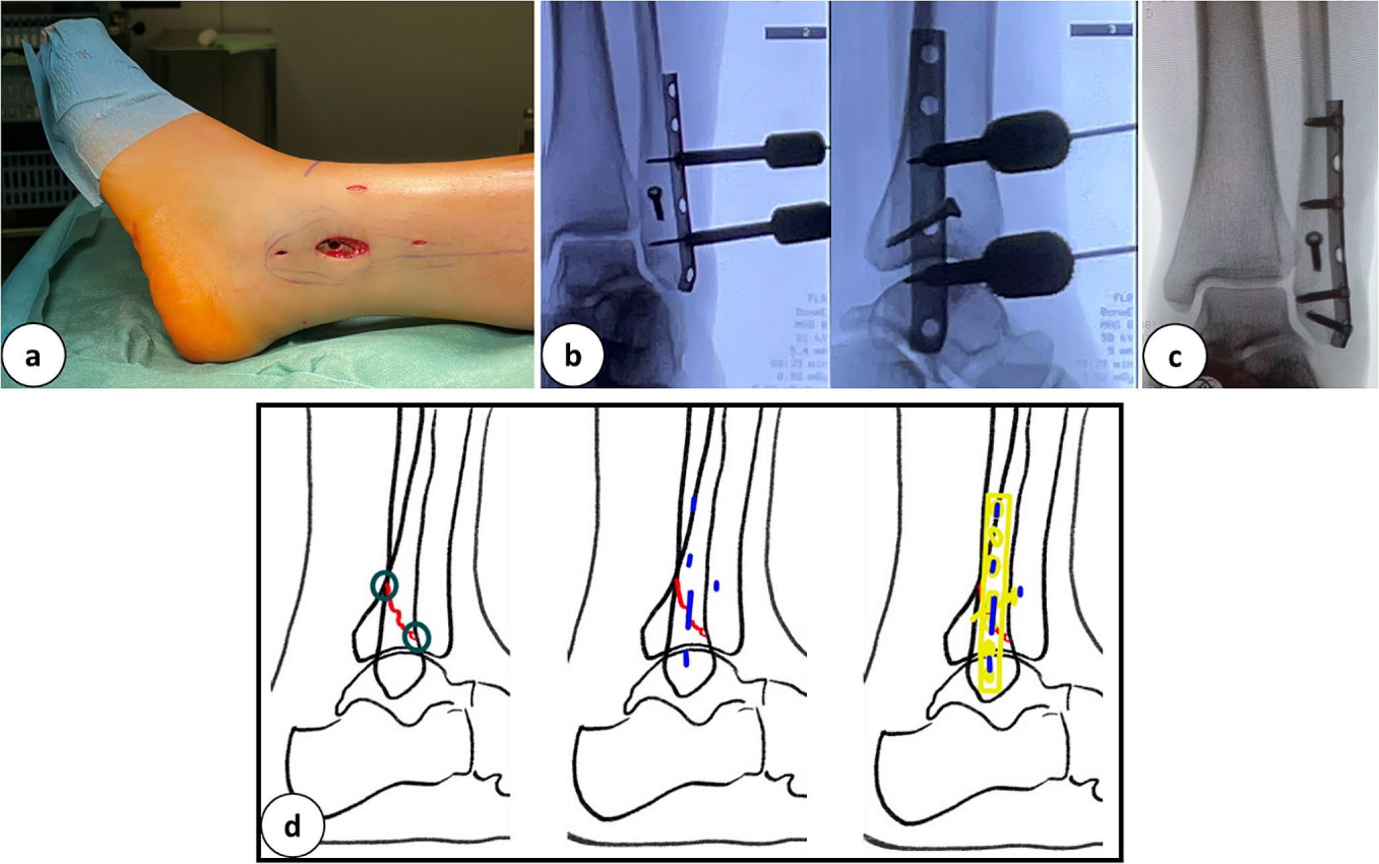
**a:** Incision directly over the left lateral malleolus for the minimal invasive plate osteosynthesis for an AO: 4F3A fracture. **b:** Intra-operative X-Ray in 2 planes after performing the lag screw and percutaneous positioning of the plate. **c:** Post-operative X-Ray in 2 planes. **d:** Schematic drawing of the fracture (red), the key landmarks, the incisions performed (blue), and the plate osteosynthesis

**Fig. 2:**
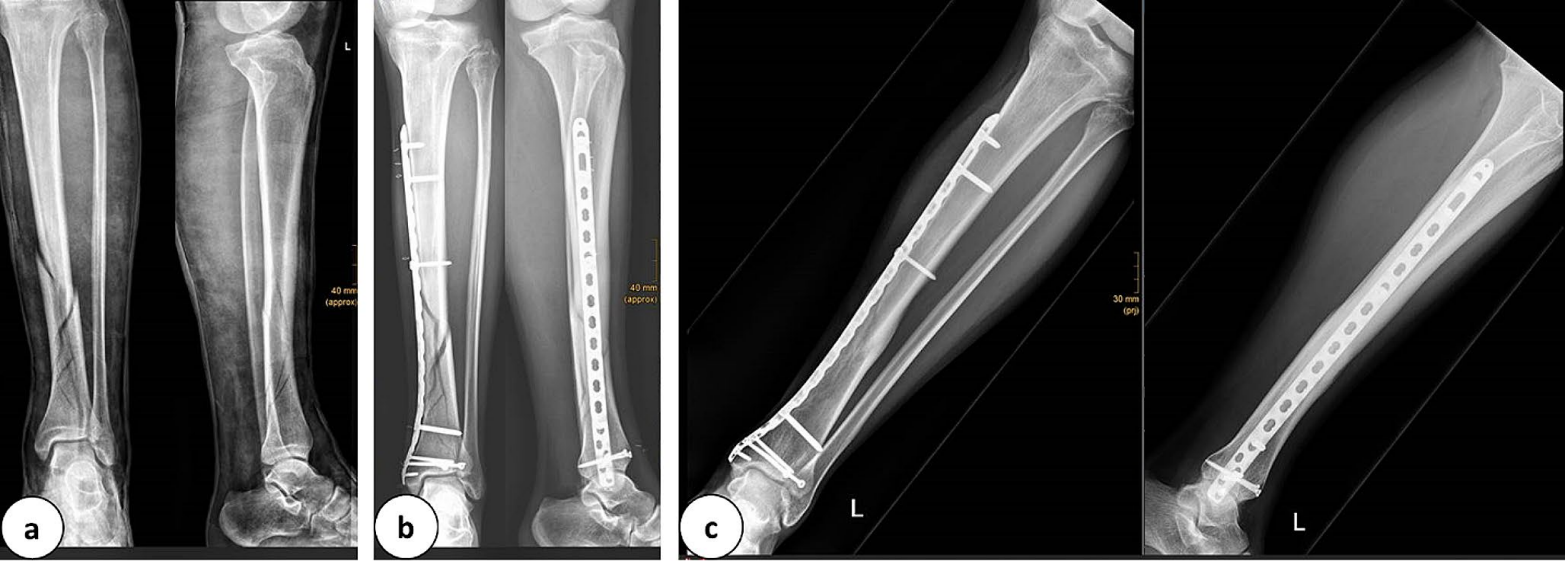
**a:** CT-scan of an olecranon fracture. **b:** Clinical pictures after marking the landmarks and performing the incisions as well as after wound closure. **c:** Lateral intra-operative X-Ray of the minimal invasive performed plate osteosynthesis

**Fig. 3:**
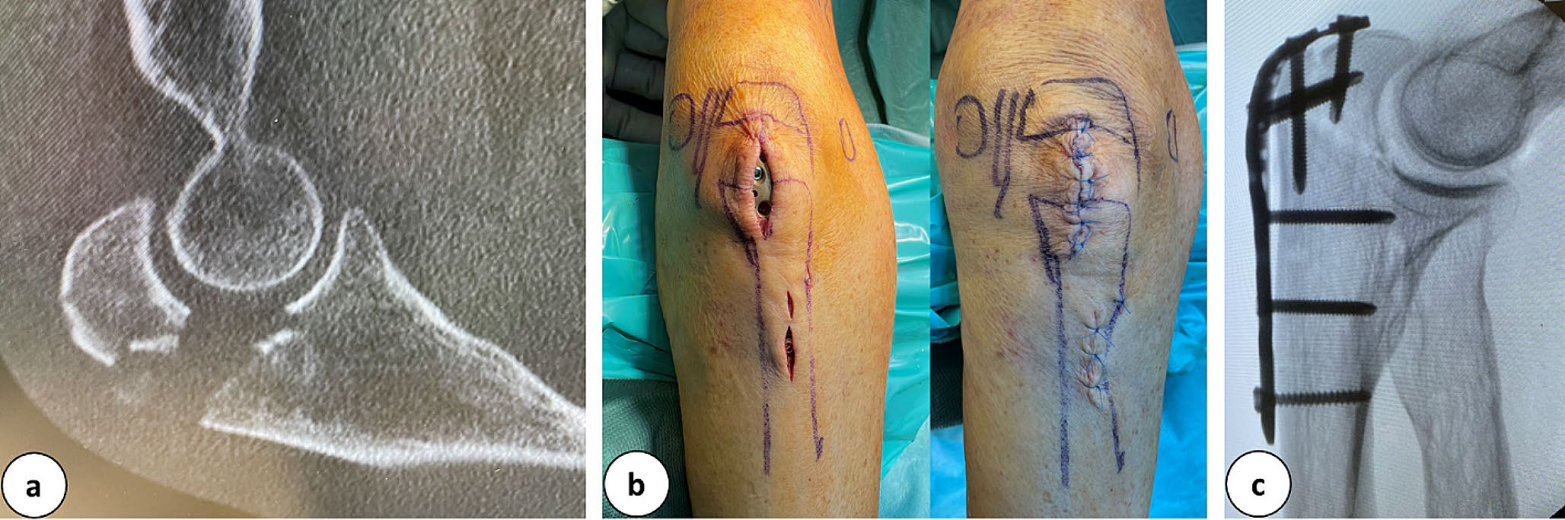
**a:** Peri-operative pictures of the minimal invasive incisions after wound closure for performing a plate osteosynthesis to stabilize a tibial head fracture (white arrow: Redon drainage, black arrow: stab incision for percutaneous locking). **b:** Intra-operative X-Ray in 2 planes after minimal invasive application of plate osteosynthesis. **c:** Schematic drawing of the tibial head fracture (red), incisions used (blue), and the plate osteosynthesis (yellow)

The “one way up” technique of plate insertion was used in all articular fractures, except for the calcaneus (97.0%) (Figs. 1, 2 and 3). The “inside up and down” technique was used mostly for non-articular fractures (13 out of 15: 86.7%), except for the mentioned calcaneus and a medial locking plate for the tibial metaphysis in a combined tibial head and shaft fracture. The two plate insertion techniques according to fracture type were summarized in Table 5.

**Table 5:** Summary of plate insertion techniques, “One way up” or “up and down” according to encountered fracture type

	Total	One Way Up	Up and Down
Articular Fractures	33	32	2
Non-Articular Fractures	24	11	13
Total	57	43	15

**Fig. 4:**
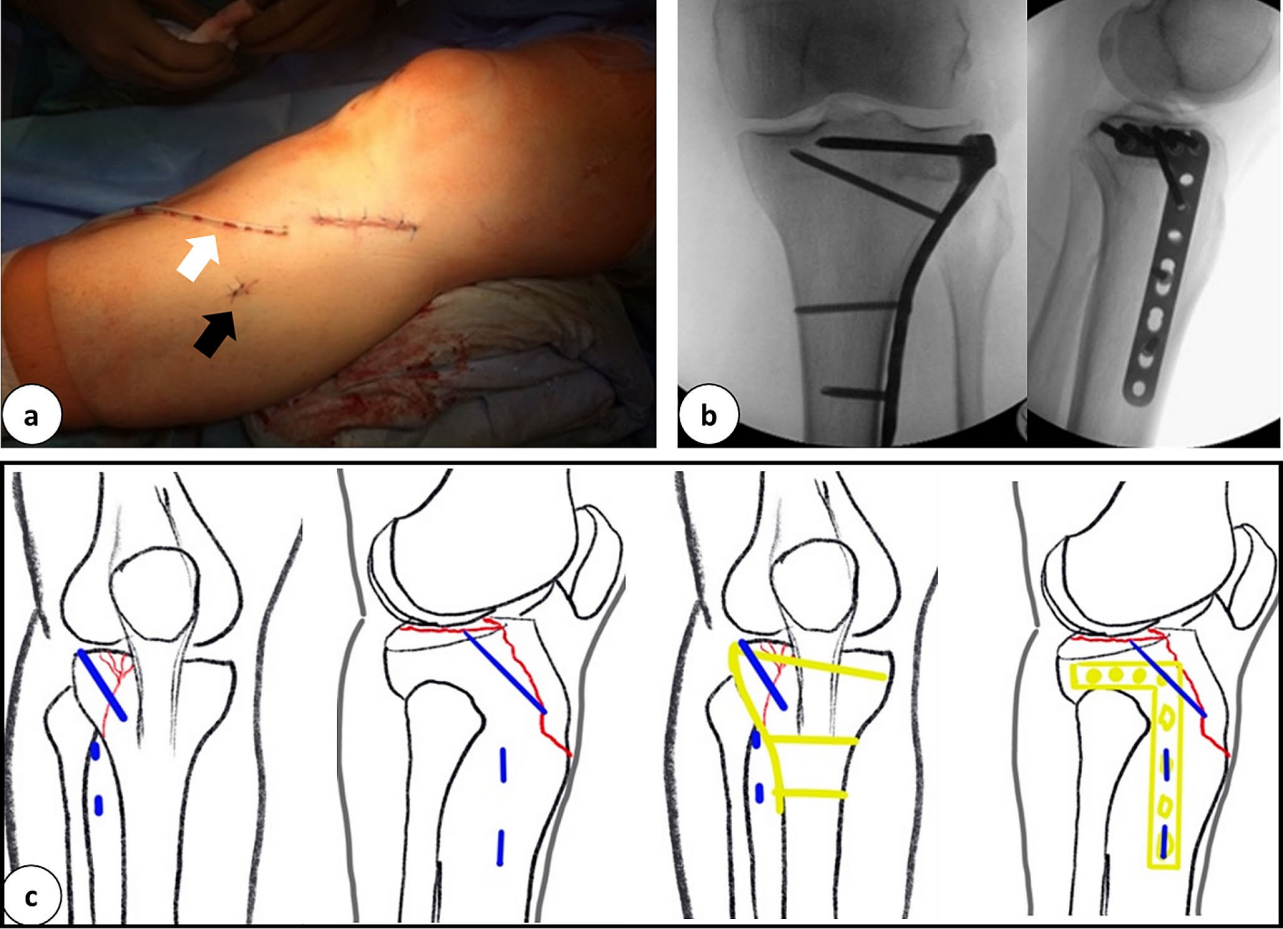
**a:** Posttraumatic X-Ray in 2 planes of a tibial shaft fracture (AO: 42C3). **b:** Post-operative X-Ray in 2 planes after performing a closed reduction and minimal invasive plate osteosynthesis. **C:** X-Ray in 2 planes 17 months post-surgical stabilization showing bony union

For non-articular shaft fractures the “inside up and down” technique was not possible because the plate was too long. Therefore, the “one way up” alternative was used (7 of 8 plate approaches; 87.5%). This included a long bone shaft fracture where the plate length almost equaled the length of the bone itself. Hence, the plate was inserted from proximal/distal and pushed all the way to down/up (Fig. 4).

Overall, 79 plates were used for fracture stabilization. This included 35 times one plate, and 22 times two plates.

Plate positioning, its feasibility as well as bony healing with its complications were summarized in Table 6.

**Table 6:** Positioning, feasibility, bony healing and complications after surgical stabilization of encountered fractures (articular/non-articular) according to the anatomical fracture location

		Positioning
Articular Fractures	Proximal Humerus	BC
	Distal Humerus	P
	Olecranon	P
	Distal Radius	S/A
	Distal Femur	S
	Proximal Tibia	S
	Calcaneus	L
Non-articular Fractures	Clavicle	BC
	Forearm	S/A
	Femoral Shaft	S/L
	Tibial Shaft	S
	Distal Tibia	S
	Malleolar (Fibula)	S

BC = Beach Chair, *P* = Prone, S = Supine, A = Arm Table, L = Lateral

Patient positioning on the operating table included: 32 times supine (10 arm tables), 7 times prone, 15 times within a beach chair, and 3 times lateral. Patients position did not differ in patients treated with ORIF alone.

If appropriate fracture control was given, the applied reduction was performed and assessed.

Four fractures healed with complications. In one case, a proximal humerus fracture treated with a locking compression plate (PHILOS) became infected, leading to the necessity of revision arthroplasty with shoulder prosthesis implantation. Another case involving a distal humeral fracture treated with two LCPs resulted in mispositioning of the ulnar LCP, leading to secondary fracture dislocation, attributed to the restricted approach of MIO. Additionally, another distal humeral fracture treated with two LCPs experienced a peri-implant fracture following a secondary trauma occurring 2 weeks post-surgery. Lastly, an 82-year-old geriatric patient expired subsequent to femoral plating, succumbing to septic urinary tract and pulmonary infections.

To illustrate the feasibility of MIO, several examples have been included in this publication.

### Example malleolar fracture, fibula

Positioning: standard supine, bump under the buttock.

Approach: directly over the fracture to control the anteroinferior fracture gap to the posterior superior border of the fibula.

Reduction: Closed (traction and supination of the foot), percutaneous pointed clamp on the distal fibula, pointed clamp on the fracture through the approach.

Fixation: Percutaneous anteroposterior lag screw and a one-third-tubular LCP.

Plate insertion: Inside up and down through the approach, percutaneous locking.

Alternatively, a sole percutaneous reduction and a one way up distal plate insertion was described [24, 25]. The direct control of the fracture was preferred to achieve anatomic reduction.

### Example tibial shaft fracture

#### Positioning: standard supine

Approach: Only for plate insertion distal over the medial malleolus.

Reduction: Closed (traction), percutaneous reduction clamp.

Fixation: Metaphyseal 3,5/4,5 mm LCP.

Plate insertion: One way up from the approach of the medial malleolus, percutaneous locking.

In case where reduction is insufficient or direct fracture control is required, an additional approach above the key landmark of the tibia can be applied.

### Example olecranon fracture

#### Positioning: standard prone

Approach: directly over the fracture to control the fracture gap from the ulnar articular surface to the radial. The control of the articular surface is achieved through the fracture.

Reduction: Closed (extension of the elbow), percutaneous K-wires to manipulate the proximal (olecranon) fragment.

Fixation: Temporary: 2 percutaneous crossed K-wires from proximal to distal. Definitive: 3,5 mm olecranon LCP.

Plate insertion: One way up to the shaft from the approach and twisting the plate ´s bend under the triceps muscle, locking through the approach in a proximal- and percutaneously distal- fashion.

### Example tibial head fracture

#### Positioning: standard supine, bump under the buttock

Approach: directly over the articular surface on the fracture site.

Reduction: Closed (varus stress), K-wires to manipulate the lateral fragment, percutaneous pointed reduction clamp (big or the ball-spiked tibial-head-reduction-clamp), raspatory tool or similar to elevate the articular surface.

Fixation:

- Temporary: K-wires from lateral to medial, inside-out medially if necessary.
- Definitive: 3,5 mm proximal tibial LCP.

Plate insertion: One way down to the shaft from the approach, locking through the approach in a proximal- and percutaneously distal- fashion.

## Discussion

Multiple MIO techniques for different anatomic regions have been described in the literature, including extensive reviews discussing its advantages and risks [8, 10, 14, 16, 18, 22–28]. Minimal invasive surgery has become widely accepted in all types of disciplines, ranging from visceral to orthopedic surgery [1–3]. New instruments permit percutaneous surgery for the spine and pelvis [29–33]. Nevertheless, to our knowledge, an attempt to generalize an algorithm with guidelines for MIO approaches has not been established yet.

The following considerations for any surgical approach to carry out an MIO need to be taken into consideration. The applied approach should include: A successful fracture reduction with instruments, adequate plate positioning and locking, as well as minimal damage to anatomical structures and tissues.

**Fig. 5:**
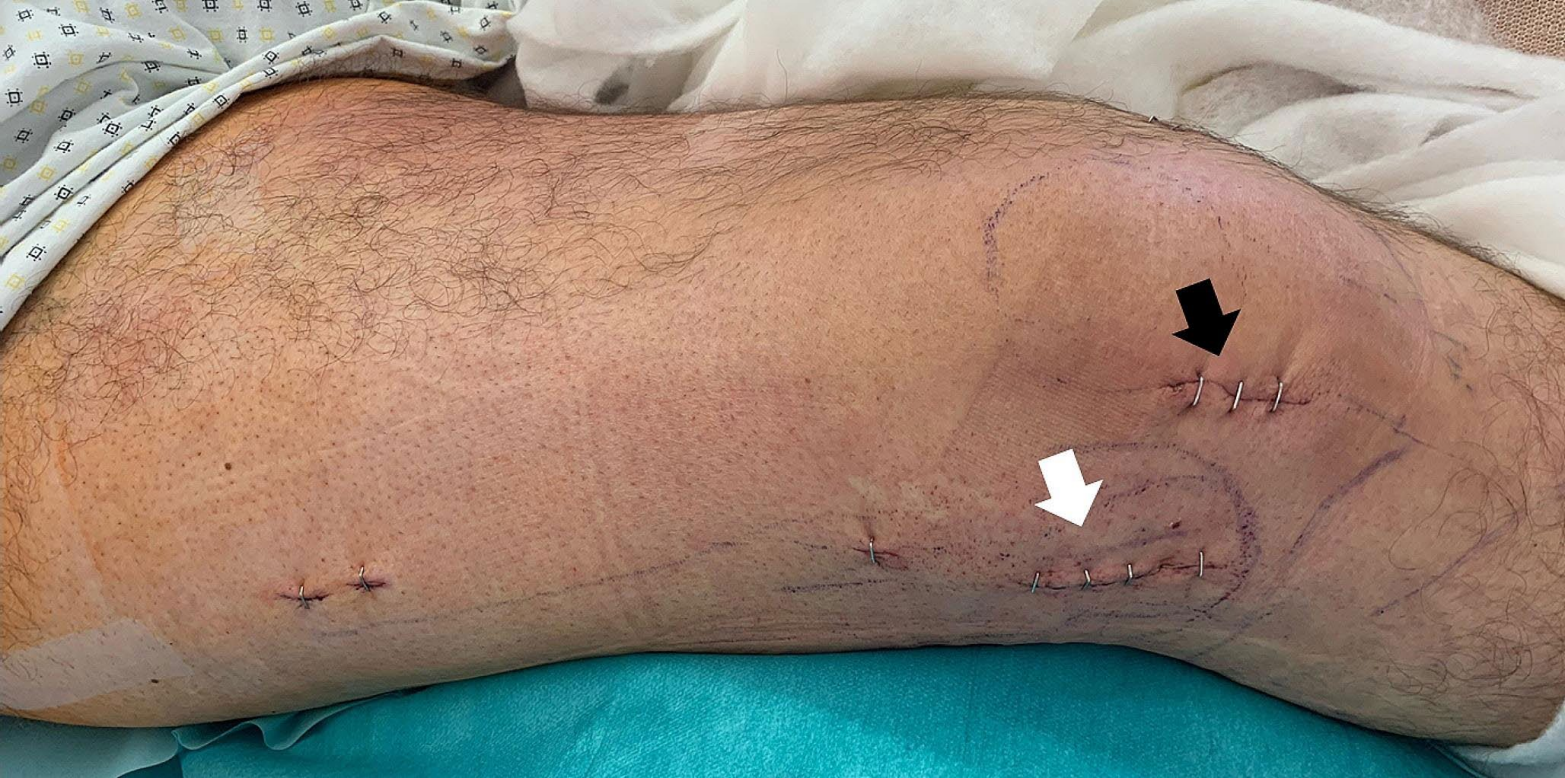
Clinical presentation of a distal femur fracture following the implementation of two distinct incisions directly over the fracture site (white arrow) and the joint (black arrow)

The surgical approach itself for articular fractures is placed directly onto the fracture and its articular surface. This permits control over both parts and the reduction of the articular surface. Due to the design of anatomic shaped locking plates, the locking of the articular segment of the plate is possible through that approach. The locking of the shaft component of the plate can be achieved through stab incisions. This surgical method was mainly used for articular fractures in this study. In some articular fractures, an additional incision was made to gain better control of the fractured articular surfaces (Fig. 5). However, it can be summarized, that 2 distinct small approaches which are placed rather deliberately, provide better fracture control than a single long one. Therefore, the soft tissue does not need to be pulled and torn.

**Fig. 6:**
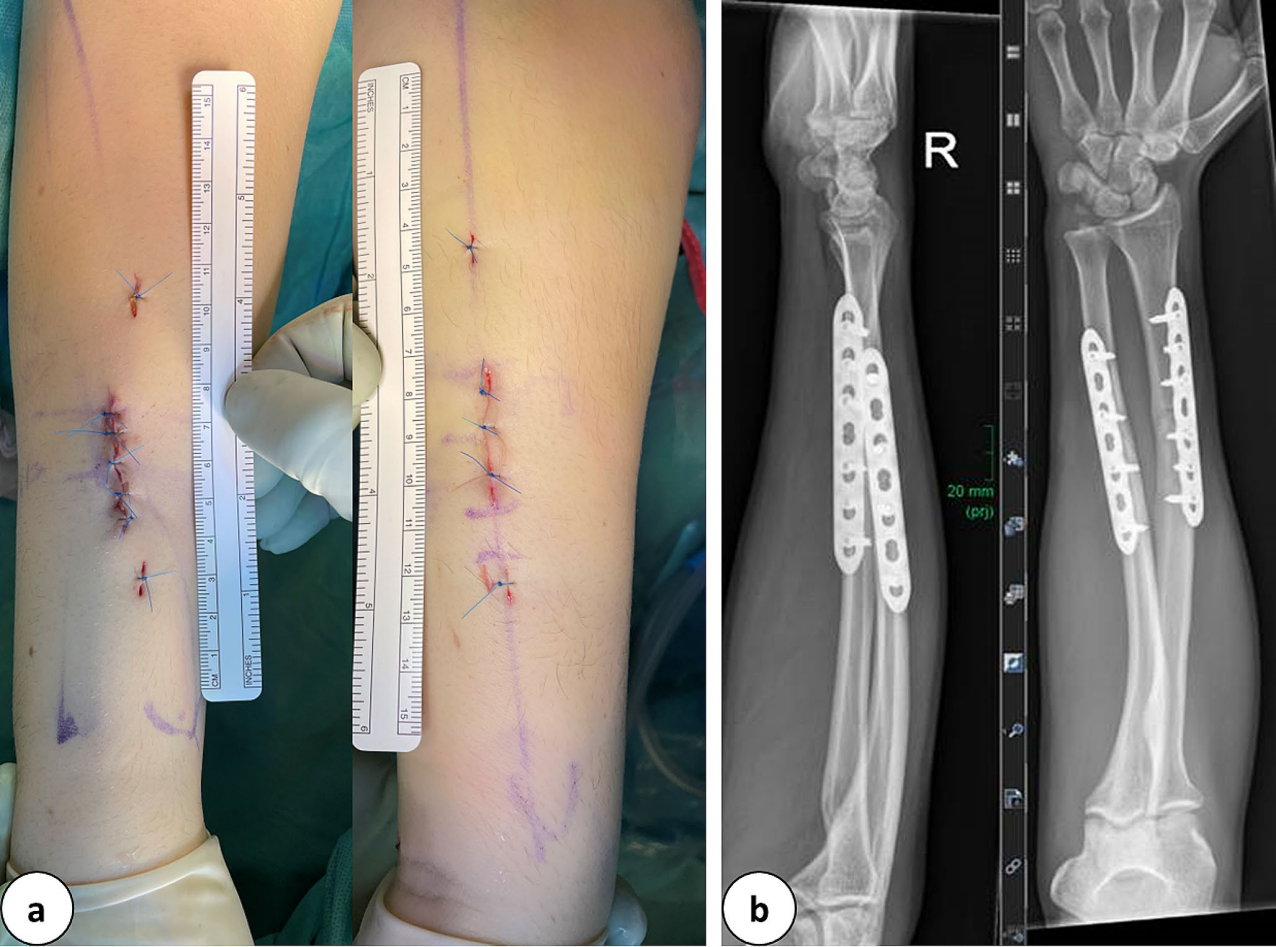
**a:** Clinical depiction of minimally invasive incisions (3.5 cm) and stab incisions executed in the scenario of a forearm fracture (AO: 2R2A3, 2U2A3). **b:** Postoperative radiographic imaging captured in two dimensions, showcasing plate osteosynthesis and fracture realignment

Most non-articular fractures required a direct approach onto the fracture and percutaneous locking of the plate proximal and distal to the fracture (Fig. 6). This permitted direct fracture control and facilitated reduction (21 out of 24 non-articular fractures).

If fracture reduction was attained through closed methods or percutaneously, the plate was positioned eccentrically, as depicted in Fig. 4. This approach to fracture reduction was employed in only 3 cases out of the 24 non-articular fractures.

The difference between these 2 methods is best described for clavicular fractures. While the most common surgical technique is a double incision without direct control of the fracture, by applying MIO approaches, a small incision is placed slightly lateral to the fracture for reduction and lateral locking, hence locking the plate medially-percutaneously [15, 34].

In our opinion, fracture control is essential for articular- and non-articular fractures. Contrary to that, most published techniques for MIO in non-articular fractures describe to direct fracture contract. Fluoroscopically controlled reduction is timely and technically inferior to open reduction and a small approach placed on a crucial landmark often suffices to optimize fracture reduction. In shaft fractures with a large fracture zone, the key landmark for reduction should be identified and the incision placed right above it.

Mainly standard approaches were used for the location of MIO surgical approaches. It permitted a fallback option for complex fractures in case reduction itself was not able to be sufficiently carried out. In such cases, an extension of the approach would permit for maximal fracture control.

Hence, in articular fractures the main incision was placed to control the articular surface and the fracture. In non-articular fractures, the incision was placed directly onto the fracture.

Closed reduction was used by applying traction and counter traction during surgery as well as adequate patient positioning on the operating table. Many techniques were combined, depending on the type of fracture to achieve an adequate reduction. For the same anatomical regions, similar techniques were used. It can be summarized that a sufficient MIO technique requires a precise planning based on imaging, the percutaneous application of reduction tools, K-wires and reduction clamps. In extensive approaches, reduction tools were usually applied directly to the bone through the main approach. This MIO reduction technique my require some training and a learning curve, especially to avoid soft tissue damage by crushing reduction clamps due to mispositioned percutaneous incisions [35]. Further, in regions with delicate anatomic structures, a thorough knowledge of their anatomic position is required to avoid accidental damage. In distal humeral fractures where the ulnar nerve is exposed in open techniques, special attention must be taken for its protection [23].

**Fig. 7:**
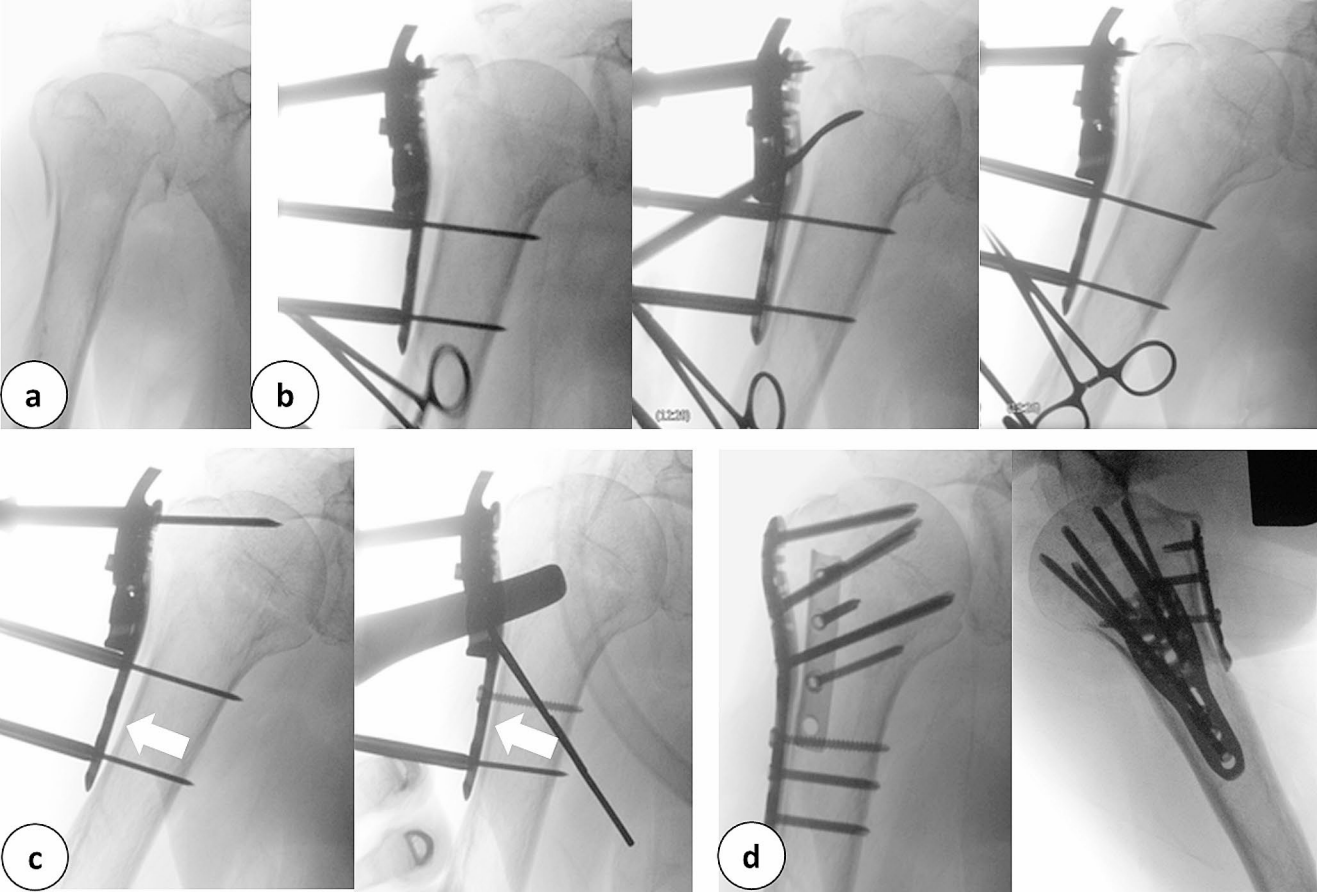
**a:** Intraoperative anterior-posterior radiograph of a multifragmentary proximal humerus fracture. **b:** Application of the stable-angle plate osteosynthesis and elevation of the dislocated humeral head with the raspatory. **c:** Fixation of the plate in the area of the humeral head and shaft with Kirschner wires and reduction of the axis by a cortical screw over the plate (white arrows). **d:** Final radiographic control with double plate osteosynthesis in two planes

In 29 cases, a plate was used as a reduction aid itself (Fig. 7). This technique seems to be specific for MIO. Compared to classical surgical sequences, in MIO the reduction and fixation can be executed simultaneously by using the plate.

In proximal humerus fractures for instance, this technique proved to be efficient, especially when using multiple drill sleeves. Tightening a plate dependent lag screw through the plate that is temporarily fixed to the bone using K-wires, reduces the greater tuberosity to the shaft and restores valgus malalignment or medial dislocation of the shaft. In tibial head fractures, the compression of the head can be applied safely using the anterolateral plate as a pressure converter, similar to washers or plates on the medial side of the tibial head. The lateral wall of the tibial head will then be realigned. An anatomic plate tightened onto the bone will reduce partially some dimensions of fracture dislocation. Applied from one side in long bones, it will reduce side-to-side and varus/valgus malalignment. Further, when the plate is centered to the bone, the ante/recurvation is corrected.

However, it can be assumed that closed reduction techniques are practically more challenging than open ones. This difference will become even more relevant for complex and comminuted fractures in anatomic regions where an anatomic reduction is necessary.

The identification of key landmarks seems important for estimating the possible reduction. The control of these landmarks permits a successful reduction. The palmar cortex for distal radius fractures or the anterior and posterior fracture borders of the fibula in malleolar fractures are such landmarks. Minimal invasive approaches for these landmarks suffice for fracture reduction and a plate can be slid in. This includes distal radius fractures, where 1,5 cm approaches were described [22]. In such cases, the locking and positioning of the plates might be more difficult than the reduction itself.

**Fig. 8:**
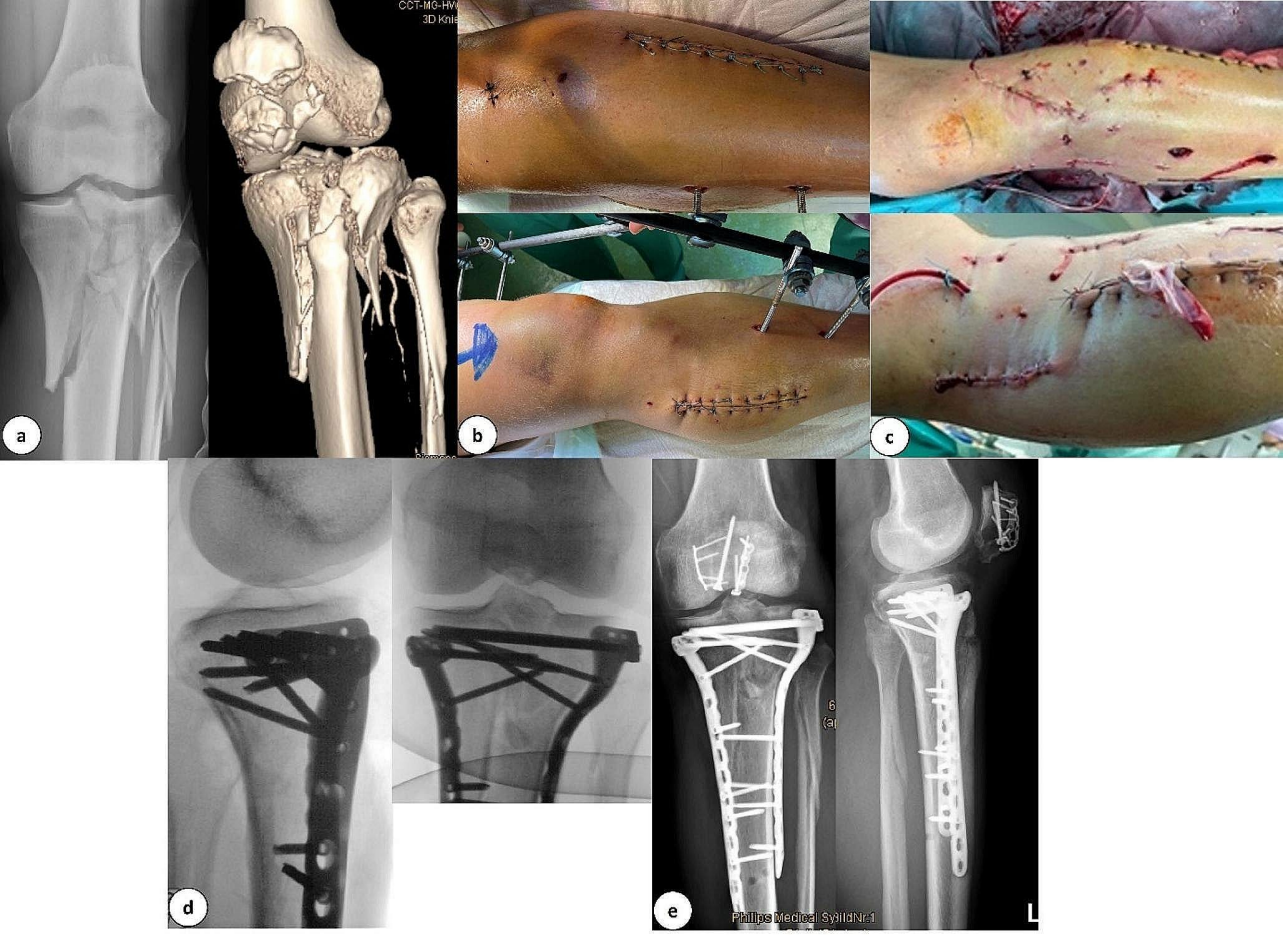
**a:** Anterior-posterior X-ray and 3-D computed tomography scan of a tibial plateau fracture (AO: 33C3.3). **b:** Preoperative clinical images before minimally invasive double plate osteosynthesis with a knee joint bridging external fixator. A condition after compartment release. **c:** Clinical images after minimally invasive double plate osteosynthesis and wound closure. **d:** Intraoperative final X-ray in 2 planes with double plate osteosynthesis. **e:** X-ray control 10 days postoperatively with double plate osteosynthesis in the area of the tibial head and miniplate with screw osteosynthesis of the patella

The condition of soft tissues is crucial to decide upon fracture treatment with MIO. In some severe open fractures with a foreseen primary delay of wound closure, an alternative and less invasive approach might reduce secondary damage to soft tissues and decrease the risk for infection (Fig. 8).

**Fig. 9:**
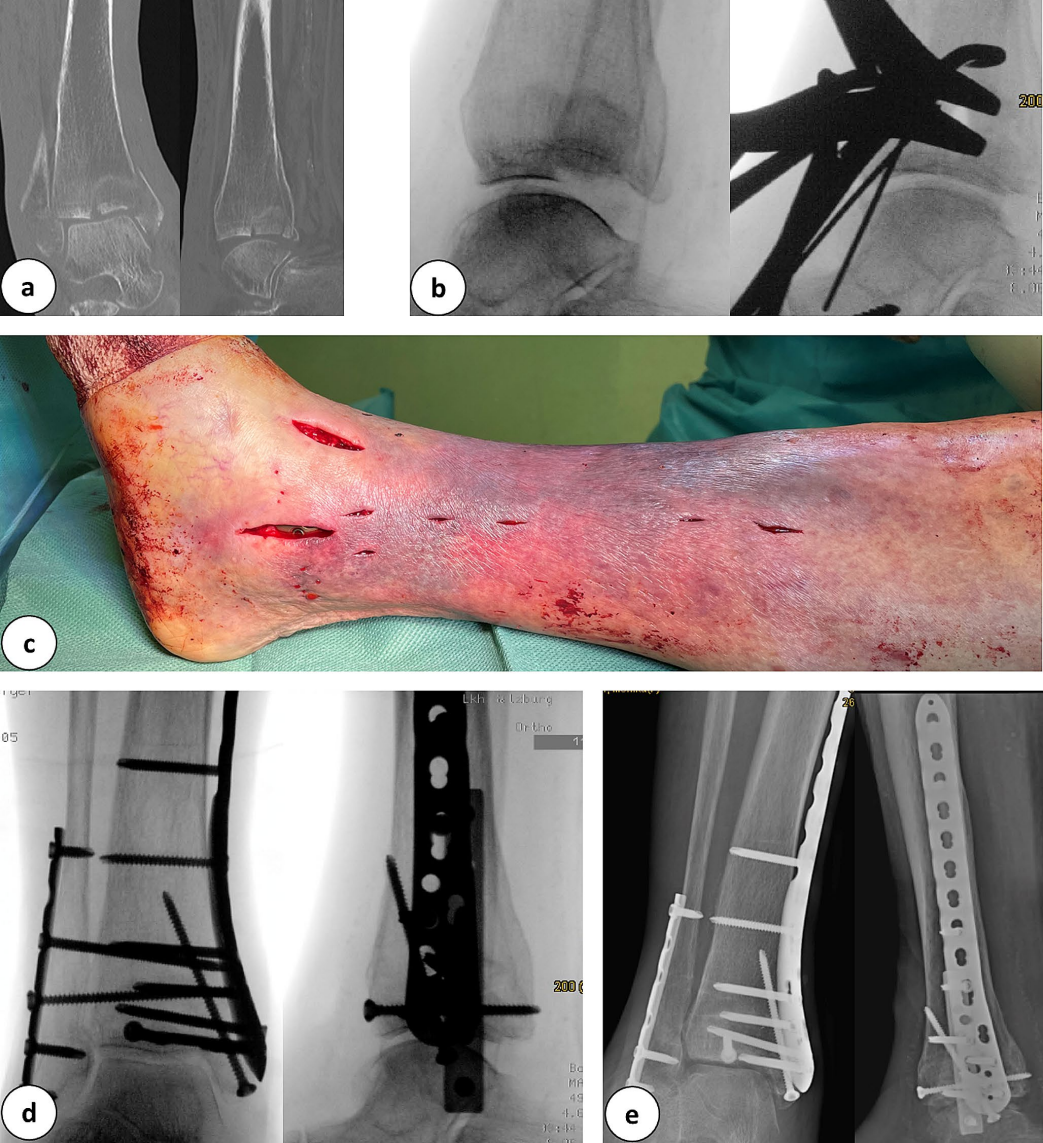
**a:** Anterior and lateral slices of a computed tomography scan of a multifragmentary distal tibial fracture with a pilon fracture. **b:** Lateral intraoperative radiographs before and after reduction of the distal tibial articular surface. **c:** Clinical images of the surgical approaches postoperatively before wound closure. **d:** Intraoperative final radiographic control after plate osteosynthesis and set screw implantation in 2 planes. **e:** Radiograph in 2 planes after removal of the set screw and fracture consolidation, 12 weeks postoperatively

In osteoporotic fractures (Fig. 9) with closed but damaged soft tissues, MIO might be a safer alternative, even when an anatomic reduction is not possible. In these cases, complex reconstruction methods for bone and soft tissues should be avoided, as their failure risk is high due to the patients age, anticoagulation, and comorbidities, such as diabetes, vascular diseases, and polyneuropathy. In some obese patients, a MIO might not be achievable at all.

It can be summarized that combined reduction techniques, percutaneous and open lead to satisfactory fracture reduction results. The plate itself plays a crucial role in the reduction technique itself [28].

The insertion of the plate is especially interesting because it used to be the determinant factor for the length of the approach. Percutaneous screw insertion and locking of the plate has become practicable through the use of drill sleeves. It permits drilling, measuring using the drill bit, and the insertion of the locking screws through stab incisions. The LISS guiding arm was one of the first implants, apart from nails, to promote this locking through stab incisions. Nevertheless, locking through stab incision is feasible with drill sleeves only without using a guiding arm. Hence, it can be concluded that the length of the approach should only be planned for reduction. In articular fractures, this can usually be implemented and plate approaches for plate insertion are mainly needed in non-articular fractures. Only 8 out of 57 fractures required a plate approach, and 7 were non-articular fractures (*n* = 24). Out of 33 articular fractures, only 1 needed a plate approach. The goal for plate insertion should be to use the existing approach for fracture reduction.

Hence, plate locking doesn’t need direct incisions other than various stab incisions.

### Limitations

Notable technical differences to non-MIOs were the percutaneous application of clamps (*n* = 9) and K-wires (*n* = 15). The application of pointed reduction clamps needed additional stab incisions for the clamp tine, and K-wire insertion required planning to avoid damage to structures at risk. Although not evaluated, adequate plate positioning is more cumbersome in MIO than in ORIF and usually needs to be verified fluoroscopically.

Other disadvantages, such as increased surgical duration and radiation exposure were widely discussed in the literature, and some benefits outweigh them. A further limitation of this study is its retrospective setup.

## Conclusion

It can be concluded that the approach should be above the articular surface and the fracture itself in articular fractures. The approach should be above the fracture in non-articular fractures. In articular fractures, the plate is inserted one way up (down), whereas in non-articular fractures the plate is inserted inside-up-and-down. Plate locking needs stab incisions only for fracture reduction, where the usual techniques need to be adapted to the limited space. The plate serves as a template for reduction. Detailed knowledge of the anatomical structures within the surgical field is essential for the safe execution of the MIO technique. Certain regions of the body are more amenable to MIO than others due to varying anatomical complexities. For instance, in the case of a distal humerus fracture, the anatomy around the elbow poses significant challenges. The proximity of critical nerves and blood vessels to the operative field necessitates a high level of precision and expertise. Therefore, MIO in this area is recommended only for highly experienced surgeons who are well-versed in the local anatomy.

Surgeons must be familiar with the “safe corridors” for screw placement and the temporary fracture reduction techniques using reduction forceps and K-wires. This ensures that the fixation devices are placed without jeopardizing surrounding neurovascular structures. Mastery of these anatomical nuances and surgical techniques is crucial to minimizing complications and achieving optimal outcomes with MIO. The standard positioning of patients on the operating table is adequate and standard approaches for incisions can be extended if necessary.

## Data Availability

Data is provided within the manuscript. Further information is available on request from the authors.
